# Single Image Super-Resolution Based on Global Dense Feature Fusion Convolutional Network

**DOI:** 10.3390/s19020316

**Published:** 2019-01-14

**Authors:** Wang Xu, Renwen Chen, Bin Huang, Xiang Zhang, Chuan Liu

**Affiliations:** State Key Laboratory of Mechanics and Control of Mechanical Structures, College of Aerospace Engineering, Nanjing University of Aeronautics and Astronautics, Nanjing 210016, Jiangsu, China; rwchen@nuaa.edu.cn (R.C.); binhuang@nuaa.edu.cn (B.H.); kalman36912@163.com (X.Z.); chuanliu@nuaa.edu.cn (C.L.)

**Keywords:** dense feature fusion, convolutional neural network, image super-resolution

## Abstract

Deep neural networks (DNNs) have been widely adopted in single image super-resolution (SISR) recently with great success. As a network goes deeper, intermediate features become hierarchical. However, most SISR methods based on DNNs do not make full use of the hierarchical features. The features cannot be read directly by the subsequent layers, therefore, the previous hierarchical information has little influence on the subsequent layer output, and the performance is relatively poor. To address this issue, a novel global dense feature fusion convolutional network (DFFNet) is proposed, which can take full advantage of global intermediate features. Especially, a feature fusion block (FFblock) is introduced as the basic module. Each block can directly read raw global features from previous ones and then learns the feature spatial correlation and channel correlation between features in a holistic way, leading to a continuous global information memory mechanism. Experiments on the benchmark tests show that the proposed method DFFNet achieves favorable performance against the state-of-art methods.

## 1. Introduction

Image super resolution, especially single image super-resolution (SISR), is a classical problem in computer version tasks. It aims to reconstruct a visually pleasing high resolution (HR) image from the degraded low resolution (LR) one. SISR has been applied in various fields, such as facial recognition, medical imaging, and surveillance systems [1,2]. The relationship between HR image and LR image is based on the situation, thus, SISR is a highly ill-posed inverse problem. A common assumption is that the LR image is a bicubic downsampled version of the HR image but, in practical application, there are so many other factors that need to be considered.

SISR methods can be roughly classified in three categories: interpolation method, reconstruction-based method, and learning-based method [3,4,5,6,7]. As deep learning (DL) becomes increasingly popular in image processing [8,9,10,11], Dong et al. [12] introduced a convolutional neural network named SRCNN, to solve SISR problems for the first time. Kim et al. [13] made a deeper network with 20 convolution layers named VDSR [13], while using skip connection to ease the training. Kim et al. [14] also proposed a deeply recursive convolutional network (DRCN) with recursive convolution layer and recursive supervision, which given help to control the amount of model parameters. 

The network performance can be furthermore improved if the module can be carefully designed. Ledig et al. [15] proposed a deeper and wider network named SRResNet [15], based on residual architecture from He et al. [16]. Tong et al. [17] introduced a dense block into SISR with a low growth rate of 16. The same dense blocks are stacked to build a deep network SRDenseNet [17] with dense skip connections. Tai et al. [18] proposed a deep network called MemNet [18] consisting of cascaded memory blocks [18] which can densely fuse global features. Hu et al. [8] proposed a cascaded multi-scale cross network (CMSC) to fuse complementary multi-scale information. Hence, the state information of some layers can be influenced not only by the adjacent information, but also by certain previous long-term information with direct connection.

As a network goes deeper, features become hierarchical since the receptive field of convolution layers in the network differs. VDSR [13], DRCN [14], SRResNet [15], SRDenseNet [17], and MemNet [18] successfully improve the performance by using the intermediate information of the network, which means the information can provide more clues to reconstruct HR image. However, none of them pay enough attention to the full use of the global features. Even though the gate unit in MemNet [18] was intended to control short-term memory and long-term memory [18] through the 1 × 1 convolution layer, it could only learn the channel correlation between features but not the feature spatial correlation. Besides, MemNet [18] interpolates LR images to get the same size as the HR images at preprocessing, and features in MemNet [18] are not directly extracted from the original LR images. 

To solve these problems, a global dense feature fusion convolutional network (DFFNet) is proposed. DFFNet can extract dense features from an original LR image to reconstruct a HR image directly, without any image scaling preprocessing. For an extremely deep network, it is not practical to extract every single layer’s output feature. A feature fusion block (FFblock) is introduced as the basic module of DFFNet. FFblock consists of a global feature fusion (GFF) unit and a feature reduction and learning (FRL) unit, which can make full use of global features, learning the feature spatial correlation and channel correlation. GFF unit concatenates all the output features of preceding blocks. The global raw features of all the preceding blocks can be directly learnt by the current block at every stage in the network. Each FFblock has a direct connection to the previous ones. Hence, a structure called global dense feature fusion (GDFF) is established by the dense feature fusion blocks (DFFBs) composed of cascaded FFblocks, where GFF unit has been densely utilized. GDFF leads to a continuous global information memory mechanism, and improves the flow of global information in the network. 

In summary, this work has three main contributions, including:

- A deep end-to-end unified framework global dense feature fusion convolutional network (DFFNet) is proposed for single image super-resolution of different scale factors. The network can learn the dense features from the original LR image and intermediate blocks and directly reconstruct HR images without any image scaling preprocessing.
- A feature fusion block (FFblock) is introduced in DFFNet, which builds a direct connection between any two blocks through global feature fusion (GFF) unit, FFblock learns the feature spatial correlation and channel correlation from the previous global features to extract higher order features.
- Dense feature fusion blocks (DFFBs) consisting of cascaded FFblocks, build global dense feature fusion so that previous global raw features can be directly learnt by the current FFblock at any stage in the network, and each FFblock in the DFFBs would adaptively decide how many of these features to be reserved, leading to a continuous global information memory mechanism.

## 2. Related Work

SISR has become a hot research topic in the field of image processing due to its wide use and great application value. The key technology of SISR is how to estimate the mapping relationship between LR image and HR image. It is essential to extract image features and perform non-linear representation to achieve high resolution image restoration.

Recently, deep learning-based methods [12,13,14,15,16,17,18,19,20,21,22] have achieved superior performance over conventional methods in SISR. SRCNN [12] firstly end-to-end learns the mapping between LR image and HR image. However, there are still existing problems, like lack of contextual connection and slow convergence. Making a network deeper and wider is the common way to improve the performance of SRCNN [12]. VDSR [13] increased the depth of the network by cascading same convolution layers while introducing residual learning to ease the difficulty of training the deep network. DRCN [14] not only utilized skip connections, but also used recursive supervision to speed up the training progress. Tai et al. [18] introduced a recursive unit based on the residual structure and a gate unit into memory block [18] to fuse the intermediate information. SRDenseNet [17] enhanced the flow of global information via dense skip connections. CMSC [19] fuses complementary multi-scale information by cascading multiple multi-scale cross modules that can learn features under different receptive fields.

However, most of these methods, such as SRCNN [12], VDSR [13], DRCN [14], and MemNet [18], need to interpolate the LR image target size, which increases the computation complexity quadratically [20]. As a result, it is hard to say those networks build an end-to-end map between an LR image and HR image without extracting the features from the original LR image. To address this problem, a transposed convolution layer was proposed by Dong et al. [20] in fast super-resolution convolutional neural networks (FSRCNN) [20], which is adopted in SRDenseNet [17] as well. Shi et al. [21] proposed an efficient subpixel convolutional neural network named ESPCN [21], directly upscaling the features into HR image. This structure was also adopted in SRResNet [15]. ESPCN [21] and FSRCNN [20] make it possible to extract features from the original LR image to reconstruct a HR image directly.

Huang et al. [22] proposed DenseNet, which introduced a dense block that let any two layers in the block have a direct connection. The same structure is also introduced in MemNet [18] and SRDenseNet [17]. More differences between MemNet [18], DenseNet [22], SRDenseNet [17], and our DFFNet will be discussed in Section 4.

The methods mentioned above have achieved state-of-art performance. However, all of them ignore the useful features in the middle of the network. Since global intermediate features are hierarchical in a very deep network, it would be helpful for SISR if the features could be fully used. To address this issue, a global dense feature fusion convolutional network is proposed to adaptively learn the global features in the intermediate layers from the LR image efficiently. The network will be detailed in the next section.

## 3. DFFNet for Image Super-Resolution

### 3.1. Basic Architecture

**Figure 1:**
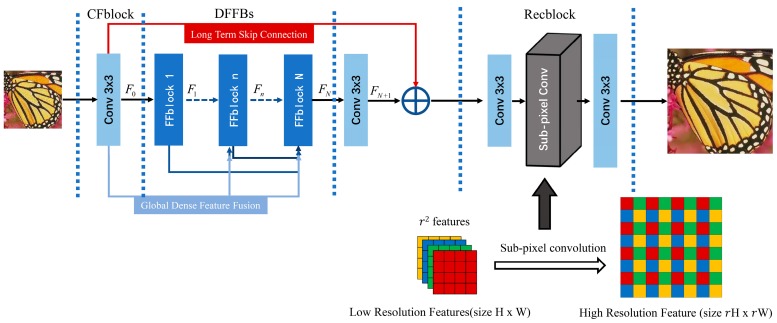
The basic architecture of the proposed dense feature fusion convolutional network (DFFNet).

The architecture of our DFFNet consists of three parts: coarse feature extraction block (CFblock), dense feature fusion blocks (DFFBs), and reconstruction block (Recblock), as shown in Figure 1. Denote ***x*** and ***y***, that represent the input and output of the network, and a convolution layer is utilized in CFblock to extract the coarse features from the LR image:

(1) $$F_{0}=f_{extract}(x)=W_{0}\times x,$$

where *f_extract_* denotes the coarse extraction function, *W*_0_ is the weight of the convolution layer, and *F*_0_ is the output of CFblock. In the DFFBs, supposing there are *N* feature fusion blocks, the output of each FFblock can be represented as

(2) $$\begin{array}{l} F_{1}=f_{FFblock_{1}}(F_{0})\\ F_{2}=f_{FFblock_{2}}(F_{0},F_{1})\\ \;\;\;\;\cdots \\ F_{n}=f_{FFblock_{n}}(F_{0},F_{1},\ldots ,F_{n-2},F_{n-1}),n\geq 2 \end{array}$$

where $f_{FFblock_{n}}$ denotes the *n*-th FFblock function, and $F_{0},F_{1},\ldots ,F_{n-2},F_{n-1}$ and $F_{n}$ are the input and output of the function, respectively. In particular, the first FFblock could only receive feed-forward features *F*_0_ from CFblock, which is illustrated in Figure 1 as well. Before Recblock, another convolution layer (Mid_conv) is stacked after DFFBs to further extract features $F_{N+1}$. $F_{N+1}$ is then added with the coarse features *F*_0_, leading to a long-term skip connection (LTSC). Experiments show that LTSC is helpful for performance improvement and training stability. At last, a structure similar to ESPCN [10] is utilized in Recblock, as shown in Figure 1. The output of DFFNet can be formulated as

(3) $$y=f_{DFFNet}(x)=f_{rec}(F_{N+1}+F_{0}),$$

where $f_{rec}$ denotes Recblock function, $f_{DFFNet}$ denotes the function of basic DFFNet.

### 3.2. Feature Fusion Block

**Figure 2:**
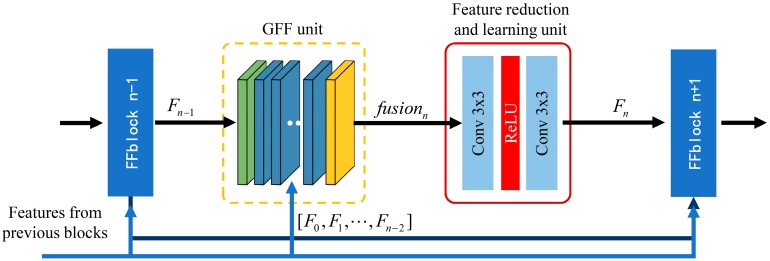
Feature fusion block. Global feature fusion (GFF) unit concatenates all the global features from previous blocks on channel dimension. Green flat cube denotes the coarse feature extraction block (CFblock) output features $F_{0}$, blue flat cubes denotes output features from previous 1, 2, …, (*n* − 2)-th FFblocks and the yellow cube denotes the output features $F_{n-1}$ from the last feature fusion block (FFblock) *n* − 1. Blue arrow denotes the flow of global information.

This section presents details about the proposed feature fusion block, as shown in Figure 2. FFblock contains two parts, including global feature fusion unit (GFF unit), and feature reduction and learning unit (FRL unit).

**Global feature fusion unit** is designed to further improve the flow of information by fusing global raw features from all the preceding FFblocks and CFblock. Global features from previous blocks are concatenated as the output of the GFF unit:

(4) $$fusion_{n}=[[F_{0},F_{1},F_{2},\ldots ,F_{n-2}],F_{n-1}],\;n\geq 2,$$

where $fusion_{n}$ is the output of GFF unit in *n*-th FFblock, $\left[F_{0},F_{1},F_{2},\ldots ,F_{n-2}\right]$ denotes the global output features of CFblock and previous 1, 2, …, (*n* − 2)-th FFblocks, and $F_{n-1}$ is the output of (*n* − 1)-th FFblock. In particular, when *n* = 1, $fusion_{1}=F_{0}$, since the first FFblock only receives feed-forward features $F_{0}$ from CFblock. If GFF unit output has $G_{F_{n}}$ feature-maps:

(5) $$G_{F_{n}}=G_{F_{0}}+G_{F_{1}}+\cdots +G_{F_{n-1}},$$

where $G_{F_{0}}$ is the number of features of CFblock output, and $G_{F_{n-1}}$ is the number of features of (*n* − 1)-th FFblock output. Each FFblock builds dense direct connections to all the subsequent ones. Therefore, by densely utilizing GFF unit, DFFBs builds global dense feature fusion (GDFF) which leads to a continuous global information memory mechanism.

**Figure 3:**
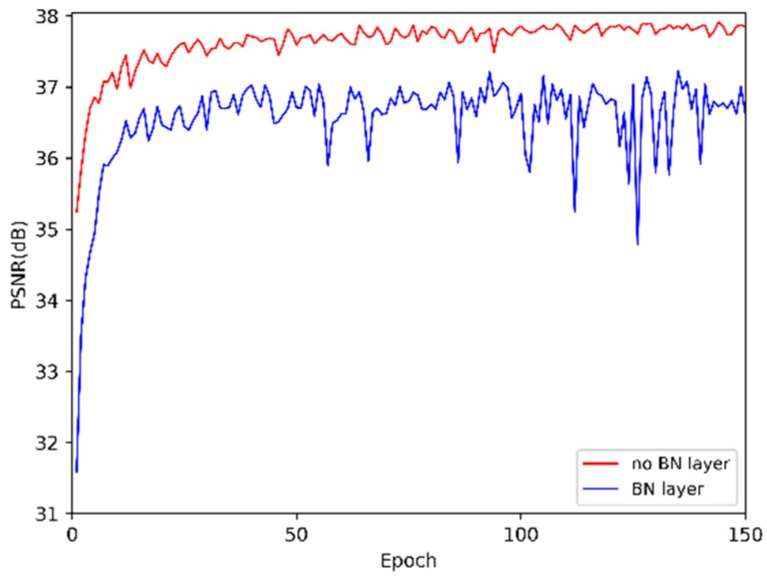
Effects of different feature reduction and learning (FRL) units added with batch normalization (BN) layer, or no BN layer, on model convergence. The curves are based on peak signal-to-noise ratio (PSNR) tested on Set5 with scale factor ×2 in 150 epochs. Other settings of model are the same as described in Section 3.4, except *N* = 16.

**Feature reduction and learning unit** is introduced to make further use of the global features and, unlike the gate unit in MemNet [18], two 3 × 3 convolution layers (C_1 and C_2) are utilized. Design of FRL unit is based on the residual structure in SRResNet [15]. Batch normalization (BN) layers are removed. As the experiment results shown in Figure 3, BN layer does not help performance improvement. Compared to 1 × 1 convolutional layer in the gate unit, the 3 × 3 convolution layers both learn the feature spatial correlation and channel correlation, then adaptively decide how much of the previous global features and feed-forward features should be reserved:

(6) $$\begin{array}{cc} F_{n} & =f_{FFblock_{n}}(F_{0,}F_{1,}\ldots ,F_{n-2},F_{n-1})\\ & =(W_{n,2}\times \sigma (W_{n,1}\times fusion_{n})) \end{array}$$

where $F_{n}$ is the output of the *n*-th FFblock, and $W_{n,1}$ and $W_{n,2}$ are the weight parameters of C_1 and C_2, respectively. σ denotes the non-linear activation function ReLU [23]. In DFFNet, the number of feed-forward features G remains the same, thus, $G_{F_{0}}=\;G_{F_{1}}=\cdots =\;G_{F_{n}}=\;G_{F_{n+1}}=G$. 

However, as the network goes forward, the number of GFF unit output features grows up, linearly. It is necessary to reduce parameters of FFblock when the network goes extremely deep. We let C_1 output have $\left[\theta G_{F_{n}}\right]$ features, where θ is the compression factor. When θ = 1, C_1 has the same number of features as GFF unit output. In our basic DFFNet, θ is set to 0.25. 

### 3.3. Reconstruction Block

As shown in Figure 1, the first 3 × 3 convolution layer (Re_conv_1) in Recblock is utilized to extract dense features. If the scale factor is r (e.g., ×2 and ×3), the output of Re_conv_1 has $2G\times r^{2}$ feature-maps. A subpixel convolution layer [21] (Re_sub_pixel_1) is stacked after Re_conv_1. Re_sub_pixel_1 is a periodic shuffling operator that rearranges the elements of a $H\times W\times C\cdot r^{2}$ tensor to a tensor of shape rH×rW×C, which is illustrated in Figure 1. The output of Re_conv_1 has 2G feature-maps with size $rH_{LR}\times rW_{LR}$, where $H_{LR}$ and $W_{LR}$ is the height and width of the input. For a large-scale factor ×4, another convolution layer (Re_conv_1_1) and subpixel convolution layer (Re_sub_pixel_2) would be stacked after Re_sub_pixel_1. The last 3 × 3 convolution layer (Re_conv_2) outputs three feature-maps, forming the reconstructed RGB image. 

### 3.4. Implementation Details

In our basic DFFNet, we set kernel size of all the convolution layers to 3 × 3, and the number of feed-forward features G remains the same, at 32, and we set zero padding to input of all layers to keep feature sizes fixed. The number of FFblocks in DFFBs is set to *N* = 32. Finally, DFFNet outputs three channel colorful images and can process gray image as well. For a detailed presentation of DFFNet, please see in Appendix A. 

Given training datasets ${\left\{img_{lr}^{\left(i\right)},img_{hr}^{\left(i\right)}\right\}}_{i=1}^{M}$, where the *M* denotes the number of image patches, $img_{hr}^{\left(i\right)}$ denotes the HR image, and $img_{lr}^{\left(i\right)}$ denotes the LR image, and *L*_1_ loss is used as training loss function:

(7) $$Loss=\frac{1}{M}\sum_{i=1}^{M}\left\|img_{hr}^{\left(i\right)}-f_{DFFNet}(img_{lr}^{\left(i\right)})\right\|$$

Although most methods use *L*_2_ loss, the *L*_1_ loss is demonstrated to be more powerful for performance and convergence [24].

## 4. Discussions

**Difference to DenseNet.** DenseNet [22] builds its architecture on the dense connections within any two layers in the dense block [22]. However, this densely connected structure is utilized only in a local way, since the size of features is different in different dense blocks, so it is impossible for the dense block to read raw features from the subsequent ones. Moreover, batch normalization (BN) layers are removed in our DFFNet, which increase computation complexity and do not help improve performance. To keep the feature sizes fixed in the network, a pooling layer is not used in DFFNet. Furthermore, feature fusion block is utilized to read the global features directly from all the preceding blocks, and learn to extract higher order features, leading to a contiguous memory mechanism which the DenseNet [22] cannot achieve.

**Difference to SRDenseNet.** The dense block in SRDenseNet [17] has the same architecture as the one in DenseNet [22]. SRDenseNet [17] introduces the dense block to solve SISR and enhance it with dense skip connections. Although the dense block can read features from the convolution layers in the block while building a local residual learning with local skip connection, the block is, however, unable to directly read global raw features from the preceding ones in a global way, like our DFFblock does. Global dense feature fusion is introduced in DFFNet, and each FFblock can learn from all the global raw features of preceding blocks and then adaptively decide how much of the current and prior information should be reserved. With full use of global raw features, DFFNet achieves better performance than SRDenseNet [17].

**Difference to MemNet.** The difference between MemNet [18] and DFFNet can be summarized in two points. First, the gate unit in memory block fuses the global features with 1 × 1 convolution layer, thus, the memory block could only learn the channel correlation between features. Two 3 × 3 convolution layers are utilized in feature fusion block (FFblock). FFblock can not only learn the channel correlation between features, but also the feature spatial correlation and, as a result, our FFblock can make further use of the intermediate features in a more global way than the memory block. Second, MemNet [18] does not directly extract features from an LR image; it has to resize the LR image with interpolation preprocessing to get the target size of the HR image, while our DFFNet extracts the features from the original LR image and utilizes Recblock to reconstruct HR image directly with dense features.

## 5. Experiments

### 5.1. Datasets and Metrics

A public high-quality dataset DVI2K [25] with 2K resolution released by Timofte et al. [25] is used for model training. DVI2K [25] includes 800 training images, 100 validation images, and 100 test images containing various types of images of landscapes, such as people, animals, insects, plants, buildings, and complex textures. The LR images used for training are obtained by bicubic downsampling with different scale factors, including ×2, ×3, and ×4, by adopting MATLAB function *imresize* with the option *bicubic* from 800 training images. Standard benchmark datasets, Set5 [26], Set14 [27], B100 [28], Urban100 [29], are used for testing. For comparison, the SISR results with different three scale factors are evaluated with peak signal-to-noise ratio (PSNR) and structural similarity (SSIM) index [30] on luminance channel (Y channel) in transformed YCbCr color space, and the same name of pixels as scale factors (×2, ×3 and ×4) are ignored, from the border, in the SISR results.

### 5.2. Training Details

For training, in each training batch, we use 16 RGB image patches with size 48 × 48 randomly cropped from LR images and the corresponding HR images for all model with different scale factors (×2, ×3, and ×4). Patches are augmented during training with random horizontal flip, vertical flip, and 90-degree rotation with random probability of 0.5. We normalize the image patches values and subtract them by the mean RGB value of the DIV2K [25] dataset as preprocessing. We implement our DFFNet with the Tensorflow framework and train the model with ADAM optimizer [31] by setting $\beta_{1}$ = 0.9, $\beta_{2}$ = 0.999, and ε = 10^−8^. The training loss function is $L_{1}$ loss. The learning rate is initialized as 0.0001 for all layers, and halved at every 200 epochs, and an epoch consists of 1000 updates. The model with different scale factors will be individually trained. It takes about 1 day with a GPU GTX1080 Ti for 300 epochs to train a basic DFFNet.

We train our model of DFFNet with scale factor ×2 (denoted as ×2 model), as described in Section 3.4, firstly, from scratch. After the ×2 model converges, we use it as a pre-trained network for the model with scale factor ×3 (denoted as ×3 model), we use ×2 model parameters to initialize all parameters in ×3 model, except the parameters in Recblock, and then fine-tune the ×3 model with the learning rate of 0.00005, about 50 epochs. The converged ×3 model will later be used as a pre-trained network for the model with scale factor ×4 (denoted as ×4 model). Training settings are kept as same as for the ×3 model.

### 5.3. Ablation Study

Table 1 presents the ablation study on the effects of global dense feature fusion (GDFF) and long-term skip connection (LTSC). Four networks in Table 1 have the same numbers of FFblocks and feed-forward features as the standard model. The baseline model (denoted as M_base) is obtained without GDFF and LTSC, based on the standard DFFNet, which has the plain structure. The performance (PSNR = 28.87 dB) of M_base is poor, even worse than Bicubic (PSNR = 33.66 dB). This is caused by the difficulty of training [1], and demonstrates that stacking many basic convolution layers does not result in better performance.

Then, we add LTSC and GDFF to M_base, resulting in M_LTSC and M_GDFF. Results show that each structure can efficiently improve the performance of M_base. This is mainly because each structure enhances the flow of information and gradient. A combination of the two structures would perform better than either in isolation. When we used two structures simultaneously (denote as M_GDFF_LTSC), DFFNet with LTSC and GDFF obviously performs the best.

**Figure 4:**
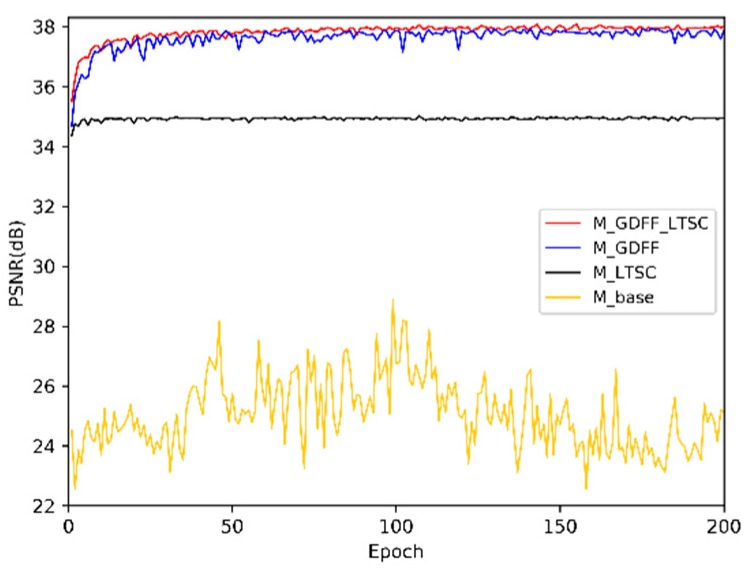
Model convergence process of different combination of GDFF and LTSC. The curves for each structure are based on average PSNR tested on Set5 with scale factor ×2 in 200 epochs. Other settings of the model are the same as described in Section 3.4.

The visualization of convergence process is presented in Figure 4. The curves verify the analyses above, and show that LTSC and GDFF both stabilize the training process while accelerating model convergence. GDFF can further improve the performance. From the red curve of M_GDFF_LTSC, we can see that LTSC can effectively reduce performance drop while improving performance, when combined with GDFF. Visual and quantitative analyses demonstrate that DFFNet can benefit greatly from LTSC and GDFF.

### 5.4. Benchmark Results

We compare our DFFNet with other methods on the benchmark testings, including Bicubic, SRCNN [12], VDSR [13], DRCN [14], SRResNet [15], LapSRN [32], CMSC [19], SRDenseNet [17], and MemNet [18]. We present quantitative results for ×2, ×3, and ×4 in Table 2. When compared with persistent models, such as MemNet [18] and SRDenseNet [17], our DFFNet performs best on all benchmarks with all scale factors. When the scale factor becomes larger (e.g., ×3, ×4), it is harder for all models to reconstruct HR images from LR images with much lower resolution, because more details need to be reconstructed. Nonetheless, our DFFNet still outperforms the others. Specifically, most images in Urban100 contain self-similar textures, although dense skip connections [6] in SRDenseNet [17] and gate unit in MemNet [18] can fuse global information to restore similar structures in images, and our DFFNet gives PSNR/SSIM of 26.20 dB/0.7893, which is 0.7 dB/0.0263 and 0.15 dB/0.0074 better than MemNet [18] and SRDenseNet [17] on Urban100 with scale factor ×4. This demonstrates that our feature fusion block (FFblock) is more effective than memory block in MemNet [18] and dense block in SRDenseNet [17], and further illustrates that fusing the global intermediate features via global dense feature fusion (GDFF) provides more clues to reconstruct HR image from the degraded image. When compared with other methods, our DFFNet still achieves the best average results on all datasets. 

We also made comparison of model complexity with other methods in Table 3. DFFNet has many more parameters than other compared methods, which would occur when the network goes deep and, so, many features need to be fused. Despite this drawback, our DFFNet is still three times faster than MemNet [18] with better performance for DFFNet, which does not need any image scaling preprocessing.

**Figure 5:**
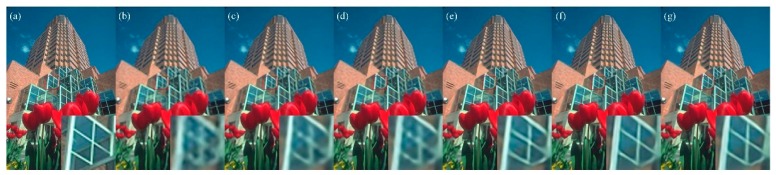
Visual results of 86,000.bmp in BSD100 processed by different methods with scale factor ×4. (**a**) Original; (**b**) SRCNN (23.38/0.7349); (**c**) DRCN (25.88/0.7776); (**d**) VDSR (25.84/0.7769); (**e**) SRDenseNet (26.21/0.7945); (**f**) MemNet (26.12/0.7967); (**g**) DFFNet (26.45/0.8041).

**Figure 6:**
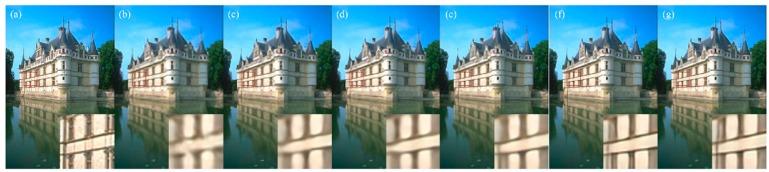
Visual results of 102061.bmp in BSD100 processed by different methods with scale factor ×4. (**a**) Original; (**b**) SRCNN (25.82/0.7730); (**c**) DRCN (26.29/0.7945); (**d**) VDSR (26.38/0.7984); (**e**) SRDenseNet (26.52/0.8064); (**f**) MemNet (26.62/0.8134); (**g**) DFFNet (26.83/0.8155).

**Figure 7:**
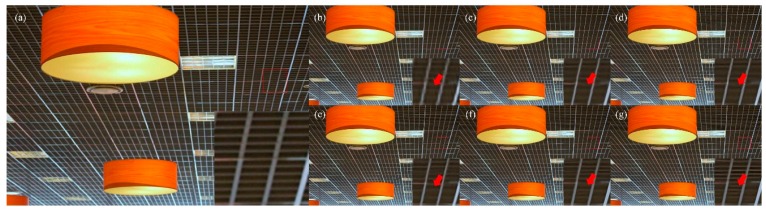
Visual results of img044.bmp in Urban100 processed by different methods with scale factor ×4. (**a**) Original; (**b**) SRCNN (29.21/0.8007); (**c**) DRCN (30.29/0.8307); (**d**) VDSR (29.77/0.8308); (**e**) SRDenseNet (30.94/0.8575); (**f**) MemNet (31.29/0.8664); (**g**) DFFNet (31.77/0.8729).

**Figure 8:**
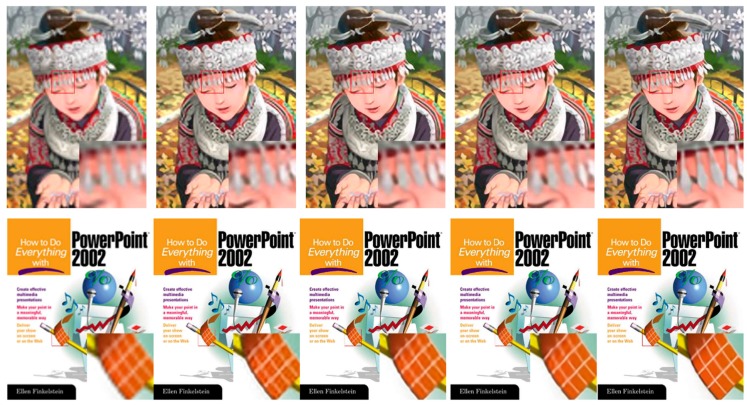
Visual results of comic.bmp and ppt3.bmp in Set14 processed by different methods with scale factor ×3. From left to right are Bicubic, SRCNN, VDSR, DRCN, and DFFNet. All compared the methods would produce obvious blur and artifacts in local areas, while our proposed method can recover clearer images with shaper edges and fewer artifacts.

Visual comparisons on scale factor ×4 are shown in Figure 5, Figure 6, Figure 7 and Figure 8. For image 86,000.bmp and 102,061.bmp, it is observed that most compared methods, such as VDSR and DRCN, would produce visible artifacts and blurred textures and edges, and even fail to recover some small textures. By contrast, our DFFNet can reconstruct clearer textures and sharper edges with fewer artifacts, closer to the original image. For the line in img044.bmp in Figure 7, as pointed out by the red arrow, all the other methods cannot successfully recover it, while our DFFNet can recover it with an obviously sharper edge. This is mainly because our DFFNet takes full advantage of global intermediate information with global dense feature fusion.

## 6. Conclusions

In this paper, we proposed a global dense feature fusion convolutional network (DFFNet) for SISR, where a feature fusion block (FFblock) is introduced as the basic module. Each FFblock can read raw features directly from all the preceding blocks in DFFNet, learning and adaptively controlling the reserve of previous global features. The global dense feature fusion (GDFF) in dense feature fusion blocks further builds the dense connections between FFblocks and coarse feature extraction block (CFblock) while stabilizing the training process and improving the flow of global information and gradient, leading to a continuous global information memory mechanism. Moreover, our DFFNet extracts features from the original LR images and reconstructs HR images with dense features directly, without any image scaling preprocessing. By fully utilizing the global features, our DFFNet leads to a deep and wide network. Quantitative and visual benchmark evaluation results demonstrate well that our DFFNet achieves superior performance over state-of-the-art methods.

## Figures and Tables

**Table 1:** Ablation study on effects of global dense feature fusion (GDFF) and long-term skip connection (LTSC). We present the best performance (average PSNR) on Set5 with scale factor ×2 in 200 epochs.

	M_Base	M_LTSC	M_GDFF	M_GDFF_LTSC
GDFF	**×**	**×**	**√**	**√**
LTSC	**×**	**√**	**×**	**√**
PSNR	28.87	35.00	37.94	38.08

**Table 2:** Public benchmark test results. Average PSNR/SSIMs for scale factor ×2, ×3, and ×4 on datasets Set5, Set14, BSD100, and Urban100.

Dataset	Scale	Bicubic	SRCNN	DRCN	SRResNet	VDSR	LapSRN	CMSC	SRDenseNet	MemNet	DFFNet
Set5	×2	33.66/0.9299	36.66/0.9542	37.63/0.9588	-/-	37.53/0.9587	37.52/0.9591	37.89/0.9605	-/-	37.78/0.9597	38.13/0.9607
	×3	30.39/0.8682	32.75/0.9090	33.82/0.9226	-/-	33.66/0.9213	33.82/0.9227	34.24/0.9266	-/-	34.09/0.9248	34.58/0.9272
	×4	28.42/0.8104	30.48/0.8628	31.53/0.8854	32.05/0.8810	31.35/0.8838	31.51/0.8855	31.91/0.8923	32.08/0.8934	31.74/0.8893	32.44/0.8949
Set14	×2	30.24/0.8688	32.42/0.9063	33.04/0.9118	-/-	33.03/0.9124	33.08/0.9130	33.41/0.9153	-/-	33.28/0.9142	33.62/0.9176
	×3	27.55/0.7742	29.28/0.8208	29.76/0.8311	-/-	29.77/0.8314	29.79/0.8320	30.09/0.8371	-/-	30.00/0.8350	30.32/0.8408
	×4	26.00/0.7027	27.49/0.7503	28.02/0.7670	28.53/0.7804	28.01/0.7674	28.19/0.7720	28.35/0.7751	28.50/0.7782	28.26/0.7723	28.65/0.7810
BSD100	×2	29.56/0.8431	31.36/0.8879	31.85/0.8942	-/-	31.90/0.8960	30.41/0.9101	32.15/0.8992	-/-	32.08/0.8978	32.29/0.9002
	×3	27.21/0.7382	28.41/0.7863	28.80/0.7963	-/-	28.82/0.7976	27.07/0.8272	29.01/0.8024	-/-	28.96/0.8001	29.21/0.8057
	×4	25.96/0.6675	26.90/0.7101	27.23/0.7233	27.57/0.7354	27.29/7251	25.21/0.7553	27.46/0.7308	27.53/0.7337	27.40/0.7281	27.76/0.7376
Urban100	×2	26.88/0.8403	29.50/0.8946	30.75/0.9133	-/-	30.76/0.9140	37.27/0.9740	31.47/0.9220	-/-	31.31/0.9195	32.32/0.9302
	×3	24.46/0.7349	26.24/0.7989	27.15/0.8276	-/-	27.14/0.8279	32.19/0.9334	27.69/0.8411	-/-	27.56/0.8376	28.25/0.8545
	×4	23.14/0.6577	24.52/0.7221	25.14/0.7510	26.07/0.7839	25.18/0.7524	29.09/0.8893	25.64/0.7692	26.05/0.7819	25.50/0.7630	26.20/0.7893

**Table 3:** Comparisons of model complexity. The results are evaluated on Set14 with scale factor ×3, where the inference speed denotes the average run time of an image in Set14.

	SRCNN	VDSR	MemNet	DFFNet (ours)
Parameters (M)	0.02	0.90	2.44	27.75
Inference speed (s)	0.004	0.03	0.369	0.113
PSNR (dB)	29.28	29.77	30.00	30.32

## Appendix A

In this section, we present the architecture of DFFNet in a more detailed way. The configuration of DFFNet has been described in Section 3.4. The number of FFblock, *N* = 32; the number of feed-forward features, *G* = 32; the compression factor, θ = 0.25. Given a preprocessed RGB image of shape 48 × 48 × 3 as input, dimensions and parameters of each block in the DFFNet have been summarized in Table A1. For the *n*-th FFblock, the output of GFF unit and C_1 have 32n and 8n feature-maps, respectively, as shown in Table A1. Before Recblock, all the outputs have the same size as the input since we have set zero padding for input of all layers to keep feature sizes fixed. We list the specifications of Recblock with a different scale factor separately in Table 3, since some parameters of Recblock depend on scale factor.

**Table A1:** Specifications for each block in DFFNet. Suppose that the input of DFFNet has the shape of 48 × 48 × 3.

CFBlock			Filters		Size			Output
			32		3 × 3			48 × 48 × 32
		GFF Unit	FRL Unit					
			C_1			C_2		
		Output	Filters	Size	Output	Filters	Size	Output
FFblock	1	48 × 48 × 32	8	3 × 3	48 × 48 × 8	32	3 × 3	48 × 48 × 32
	2	48 × 48 × 64	16	3 × 3	48 × 48 × 16	32	3 × 3	48 × 48 × 32
	3	48 × 48 × 96	24	3 × 3	48 × 48 × 24	32	3 × 3	48 × 48 × 32
	4	48 × 48 × 128	32	3 × 3	48 × 48 × 32	32	3 × 3	48 × 48 × 32
	5	48 × 48 × 160	40	3 × 3	48 × 48 × 40	32	3 × 3	48 × 48 × 32
	6	48 × 48 × 192	48	3 × 3	48 × 48 × 48	32	3 × 3	48 × 48 × 32
	7	48 × 48 × 224	56	3 × 3	48 × 48 × 56	32	3 × 3	48 × 48 × 32
	8	48 × 48 × 256	64	3 × 3	48 × 48 × 64	32	3 × 3	48 × 48 × 32
	9	48 × 48 × 288	72	3 × 3	48 × 48 × 72	32	3 × 3	48 × 48 × 32
	10	48 × 48 × 320	80	3 × 3	48 × 48 × 80	32	3 × 3	48 × 48 × 32
	11	48 × 48 × 352	88	3 × 3	48 × 48 × 88	32	3 × 3	48 × 48 × 32
	12	48 × 48 × 384	96	3 × 3	48 × 48 × 96	32	3 × 3	48 × 48 × 32
	13	48 × 48 × 416	104	3 × 3	48 × 48 × 104	32	3 × 3	48 × 48 × 32
	14	48 × 48 × 448	112	3 × 3	48 × 48 × 112	32	3 × 3	48 × 48 × 32
	15	48 × 48 × 480	120	3 × 3	48 × 48 × 120	32	3 × 3	48 × 48 × 32
	16	48 × 48 × 512	128	3 × 3	48 × 48 × 128	32	3 × 3	48 × 48 × 32
	17	48 × 48 × 544	136	3 × 3	48 × 48 × 136	32	3 × 3	48 × 48 × 32
	18	48 × 48 × 576	144	3 × 3	48 × 48 × 144	32	3 × 3	48 × 48 × 32
	19	48 × 48 × 608	152	3 × 3	48 × 48 × 152	32	3 × 3	48 × 48 × 32
	20	48 × 48 × 640	160	3 × 3	48 × 48 × 160	32	3 × 3	48 × 48 × 32
	21	48 × 48 × 672	168	3 × 3	48 × 48 × 168	32	3 × 3	48 × 48 × 32
	22	48 × 48 × 704	176	3 × 3	48 × 48 × 176	32	3 × 3	48 × 48 × 32
	23	48 × 48 × 736	184	3 × 3	48 × 48 × 184	32	3 × 3	48 × 48 × 32
	24	48 × 48 × 768	192	3 × 3	48 × 48 × 192	32	3 × 3	48 × 48 × 32
	25	48 × 48 × 800	200	3 × 3	48 × 48 × 200	32	3 × 3	48 × 48 × 32
	26	48 × 48 × 832	208	3 × 3	48 × 48 × 208	32	3 × 3	48 × 48 × 32
	27	48 × 48 × 864	216	3 × 3	48 × 48 × 216	32	3 × 3	48 × 48 × 32
	28	48 × 48 × 896	224	3 × 3	48 × 48 × 224	32	3 × 3	48 × 48 × 32
	29	48 × 48 × 928	232	3 × 3	48 × 48 × 232	32	3 × 3	48 × 48 × 32
	30	48 × 48 × 960	240	3 × 3	48 × 48 × 240	32	3 × 3	48 × 48 × 32
	31	48 × 48 × 992	248	3 × 3	48 × 48 × 248	32	3 × 3	48 × 48 × 32
	32	48 × 48 × 1024	256	3 × 3	48 × 48 × 256	32	3 × 3	48 × 48 × 32
Mid_conv			filters		size			output
			32		3 × 3			48 × 48 × 32
For scale factor ×2								
Recblock	Re_conv_1		filters		size			output
			256		3 × 3			48 × 48 × 256
	Re_sub_pixel		filters		size			output
			/		/			96 × 96 × 64
	Re_conv_2		filters		size			output
			3		3 × 3			96 × 96 × 3
For scale factor ×3								
Recblock	Re_conv_1		filters		size			output
			576		3 × 3			48 × 48 × 576
	Re_sub_pixel		filters		size			output
			/		/			144 × 144 × 64
	Re_conv_2		filters		size			output
			3		3 × 3			144 × 144 × 3
For scale factor ×4								
Recblock	Re_conv_1_1		filters		size			output
			256		3 × 3			48 × 48 × 256
	Re_sub_pixel_1		filters		size			output
			/		/			96 × 96 × 64
	Re_conv_1_2		filters		size			output
			256		3 × 3			96 × 96 × 256
	Re_sub_pixel_2		filters		size			output
			/		/			192 × 192 × 64
	Re_conv_2		filters		size			output
			3		3 × 3			192 × 192 × 3

## References

[1] Ziwei L., Chengdong W., Dongyue C., Yuanchen Q., Chunping W. Overview on image super resolution reconstruction. Proceedings of the IEEE Control and Decision Conference.

[2] Xu S., Xiao-Guang L., Jia-Feng L., Li Z. (2017). Review on Deep Learning Based Image Super-resolution Restoration Algorithms. Acta Autom. Sin..

[3] Timofte R., Smet V.D., Gool L.V. A+: Adjusted Anchored Neighborhood Regression for Fast Super-Resolution. Proceedings of the Asian Conference on Computer Vision.

[4] Jiang J., Ma X., Chen C., Lu T., Wang Z., Ma J. (2017). Single Image Super-Resolution via Locally Regularized Anchored Neighborhood Regression and Nonlocal Means. IEEE Trans. Multimedia.

[5] Chen C. (2016). Noise Robust Face Image Super-Resolution through Smooth Sparse Representation. IEEE Trans. Cybern..

[6] Zhu Z., Guo F., Yu H., Chen C. (2014). Fast single image super-resolution via self-example learning and sparse representation. IEEE Trans. Multimedia.

[7] Chen C., Fowler J.E. Single-image super-resolution using multihypothesis prediction. Proceedings of the 2012 IEEE Conference Record of the Forty Sixth Asilomar Conference on Signals, Systems and Computers (ASILOMAR).

[8] Jin Y., Kuwashima S., Kurita T. (2017). Fast and Accurate Image Super Resolution by Deep CNN with Skip Connection and Network in Network. Proceedings of the International Conference on Neural Information Processing.

[9] Lin G., Milan A., Shen C., Reid I. (2016). RefineNet: Multi-Path Refinement Networks for High-Resolution Semantic Segmentation. arXiv.

[10] Chen L.C., Papandreou G., Schroff F., Adam H. (2017). Rethinking Atrous Convolution for Semantic Image Segmentation. arXiv.

[11] Szegedy C., Ioffe S., Vanhoucke V., Alemi A.A. (2016). Inception-v4, Inception-ResNet and the Impact of Residual Connections on Learning. arXiv.

[12] Dong C., Loy C.C., He K., Tang X. Learning a deep convolutional network for image super-resolution. Proceedings of the ECCV 2014.

[13] Kim J., Lee J.K., Lee K.M. Accurate image super-resolution using very deep convolutional networks. Proceedings of the CVPR 2016.

[14] Kim J., Le J.K., Le K.M. Deeply-recursive convolutional network for image super resolution. Proceedings of the IEEE Conference on Computer Vision and Pattern Recognition.

[15] Ledig C., Theis L., Huszar F., Caballero J., Cunningham A., Acosta A., Aitken A., Tejani A., Totz J., Wang Z. (2016). Photo-realistic single image super-resolution using a generative adversarial network. arXiv.

[16] He K., Zhang X., Ren S., Sun J. Deep Residual Learning for Image Recognition. Proceedings of the IEEE Conference on Computer Vision and Pattern Recognition.

[17] Tong T., Li G., Liu X., Gao Q. Image Super-Resolution Using Dense Skip Connections. Proceedings of the 2017 IEEE International Conference on Computer Vision (ICCV).

[18] Tai Y., Yang J., Liu X., Xu C. MemNet: A Persistent Memory Network for Image Restoration. Proceedings of the IEEE International Conference on Computer Vision.

[19] Hu Y., Gao X., Li J., Huang Y., Wang H. (2018). Single Image Super-Resolution via Cascaded Multi-Scale Cross Network. arXiv.

[20] Dong C., Loy C.C., Tang X. Accelerating the Super-Resolution Convolutional Neural Network. Proceedings of the European Conference on Computer Vision 2016.

[21] Shi W., Caballero J., Huszár F., Totz J., Aitken A.P., Bishop R., Rueckert D., Wang Z. Real-time single image and video super-resolution using an efficient sub-pixel convolutional neural network. Proceedings of the CVPR 2016.

[22] Huang G., Liu Z., Van Der Maaten L., Weinberger K.Q. Densely Connected Convolutional Networks. Proceedings of the CVPR 2017 Workshops.

[23] Glorot X., Bordes A., Bengio Y. Deep sparse rectifier neural networks. Proceedings of the AISTATS 2011.

[24] Dai D., Timofte R., Gool L.V. (2015). Jointly Optimized Regressors for Image Super-resolution. Comput. Gr. Forum.

[25] Timofte R., Agustsson E., Van Gool L., Yang M.H., Zhang L., Lim B., Son S., Kim H., Nah S., Lee K.M. Ntire 2017 challenge on single image super-resolution: Methods and results. Proceedings of the CVPR 2017 Workshops.

[26] Bevilacqua M., Roumy A., Guillemot C., Alberi-Morel M.L. Low-complexity single-image super-resolution based on nonnegative neighbor embedding. Proceedings of the BMVC 2012.

[27] Zeyde R., Elad M., Protter M. On single image scale-up using sparse-representations. Proceedings of the International Conference on Curves and Surfaces.

[28] Martin D., Fowlkes C., Tal D., Malik J. A database of human segmented natural images and its application to evaluating segmentation algorithms and measuring ecological statistics. Proceedings of the ICCV 2001.

[29] Huang J.-B., Singh A., Ahuja N. Single image super resolution from transformed self-exemplars. Proceedings of the CVPR 2015.

[30] Wang Z., Bovik A.C., Sheikh H.R., Simoncelli E.P. (2004). Image quality assessment: From error visibility to structural similarity. IEEE Trans. Image Process..

[31] Kingma D., Ba J. Adam: A method for stochastic optimization. Proceedings of the ICLR 2015.

[32] Lai W.S., Huang J.B., Ahuja N., Yang M.H. (2017). Deep Laplacian Pyramid Networks for Fast and Accurate Super-Resolution. arXiv.

